# Trends in central precocious puberty incidence in Japan during the COVID-19 pandemic

**DOI:** 10.3389/fped.2026.1769902

**Published:** 2026-03-03

**Authors:** Yosuke Komatsu, Nobuyuki Kikuchi, Kuniyuki Nishiyama, Koji Ohsugi, Kentaro Shiga

**Affiliations:** 1 Department of Pediatrics, Yokohama Rosai Hospital, Japan Organization of Health and Safety, Yokohama, Japan; 2 Children's Medical Center, Yokohama City University Medical Center, Yokohama, Japan; 3 Department of Pediatrics, Yokohama City University, Yokohama, Japan; 4 Department of Pediatrics, Yokohama Medical Center, National Hospital Organization, Yokohama, Japan; 5 Department of Pediatrics, Odawara Municipal Hospital, Odawara, Japan

**Keywords:** central precocious puberty, childhood obesity, COVID-19 pandemic, epidemiology, incidence trends, pediatric endocrinology

## Abstract

**Background:**

The incidence of central precocious puberty (CPP) has reportedly increased worldwide during the coronavirus disease 2019 (COVID-19) pandemic; however, multicenter data from Japan remain limited. This study aimed to evaluate temporal changes in the incidence and clinical characteristics of CPP before, during, and after the pandemic.

**Methods:**

We conducted a multicenter retrospective observational study across four pediatric endocrinology centers in Kanagawa, Japan. Newly diagnosed CPP cases from 2018 to 2023 were categorized into three periods: pre-pandemic (2018–2019), pandemic (2020–2021), and post-pandemic (2022–2023). Incidence rate ratios (IRRs) were calculated using quasi-Poisson regression, with population size included as an offset. Clinical characteristics—including age at diagnosis, bone age, degree of overweight, and hormone profiles—were compared across periods using the Kruskal–Wallis test.

**Results:**

A total of 118 children (94 girls and 24 boys) were diagnosed with CPP during the study period. Among girls, CPP incidence increased significantly during the pandemic compared with the pre-pandemic period [IRR 2.47; 95% confidence interval (CI): 1.29–5.03]. In boys, incidence also increased with a statistically significant IRR; however, the estimate was accompanied by wide confidence intervals owing to the small number of cases. Elevated incidence rates in girls persisted into the post-pandemic period. No significant differences were observed across periods in age at diagnosis, degree of bone age advancement, degree of overweight, or basal and stimulated hormone levels. Nevertheless, the cohort consistently exhibited higher degrees of overweight compared with national reference values.

**Conclusions:**

This multicenter study demonstrates a significant increase in CPP incidence among girls during the COVID-19 pandemic in Japan, with sustained elevation in the post-pandemic period. Although clinical characteristics remained largely unchanged, the consistently higher degree of overweight underscores the need to consider lifestyle and environmental factors that may have been exacerbated during the pandemic. Ongoing surveillance and reevaluation of CPP diagnostic criteria may be warranted to address emerging epidemiological trends.

## 1 Introduction

Central precocious puberty (CPP), defined as the early onset of puberty resulting from premature activation of the hypothalamic–pituitary–gonadal (HPG) axis (1), is typically characterized by the development of secondary sexual characteristics—such as breast development in girls or testicular enlargement in boys—at an age significantly earlier than population norms (2). Although diagnostic thresholds vary across countries and clinical guidelines, early identification and appropriate management of CPP remain essential to prevent compromised adult height and potential psychosocial consequences.

An association between the coronavirus disease 2019 (COVID-19) pandemic and an increased incidence of CPP was first reported in Italy in 2020 (3). Subsequently, multiple studies from different regions have described similar trends, particularly among girls. These findings have been comprehensively summarized in several recent review articles (4–6), suggesting that pandemic-related lifestyle changes, psychosocial stress, and altered environmental exposures may have contributed to earlier pubertal onset. In contrast, evidence regarding CPP incidence among boys has been inconsistent, with several studies and reviews reporting no clear or uniform increase during the pandemic.

In Japan, evidence remains limited. To date, only one single-center study has examined this issue, demonstrating a significant increase in CPP incidence during the pandemic compared with the pre-pandemic period (7). However, the lack of multicenter data restricts the ability to assess regional variation, evaluate broader epidemiological patterns, and contextualize Japan's trends within the international landscape.

To address this knowledge gap, we conducted a multicenter retrospective observational study across four tertiary pediatric endocrinology centers in Kanagawa Prefecture, Japan. This study aimed to clarify temporal trends in CPP incidence before, during, and after the COVID-19 pandemic and to assess whether clinical characteristics differed across these periods.

## 2 Materials and methods

### 2.1 Study design and setting

This multicenter retrospective observational study was conducted at four hospitals in Kanagawa Prefecture, Japan: Yokohama Rosai Hospital (Department of Pediatrics), Yokohama Medical Center, Odawara Municipal Hospital, and Yokohama City University Medical Center. All participating institutions are secondary or tertiary care centers providing outpatient pediatric endocrinology services. During the pandemic period, Japan did not implement mandatory lockdowns as seen in some other countries. Instead, the government declared states of emergency, during which residents were strongly encouraged to limit non-essential outings. Schools experienced intermittent closures or operational restrictions, and many social and recreational activities were curtailed. These measures were implemented uniformly across Kanagawa Prefecture.

### 2.2 Ethical approval

This study was approved by the Ethics Committee of Yokohama Rosai Hospital (Approval ID: 2023-061). Owing to the retrospective study design, the requirement for individual informed consent was waived. In accordance with institutional and national ethical guidelines, an opt-out procedure was implemented to allow patients and their families to decline participation. Study information was publicly posted on the hospital website, and individuals could request exclusion from the dataset.

### 2.3 Participants

Eligible participants were children who first presented to one of the participating hospitals between January 2018 and December 2023 and were diagnosed with CPP at their initial evaluation. Diagnosis followed the official Japanese diagnostic criteria outlined in the *Guidelines for the Diagnosis and Treatment of Pediatric Endocrine Disorders* (8). These criteria incorporate clinical evidence of early pubertal onset, advanced bone age, activation of the HPG axis (elevated gonadotropins and sex steroids), and exclusion of peripheral precocious puberty or other etiologies (7).

Primary diagnostic findings include age-inappropriate sexual maturation (e.g., breast development in girls and testicular enlargement in boys), whereas secondary findings include accelerated growth and markedly advanced bone age. A diagnosis of CPP was made when patients met either: (i) at least two primary findings, or (ii) one primary finding and one secondary finding, together with supportive laboratory results and exclusion criteria. In exceptional cases, children exceeding age thresholds by up to one year were diagnosed if all other criteria were fulfilled and if their pre-onset growth curve had been below −1 standard deviation (SD).

Patients were excluded if they had a prior diagnosis or treatment for CPP at another facility or if they were diagnosed with peripheral precocious puberty. Eligible patients were categorized into three groups based on the timing of their initial visit: Pre-pandemic (January 2018–December 2019), Pandemic (January 2020–December 2021), and Post-pandemic (January 2022–December 2023). The post-pandemic period was operationally defined as the time following the lifting of major pandemic-related social restrictions in Japan, rather than implying a complete resolution of pandemic-related influences.

The pre-pandemic period was limited to the two years immediately preceding the COVID-19 pandemic to ensure consistency in diagnostic practices and data availability across all participating institutions. Although a longer pre-pandemic observation period might have further clarified underlying secular trends, such data were not uniformly available across all centers.

### 2.4 Data collection

Clinical data were retrospectively extracted from electronic medical records for all eligible patients. Variables included the date of initial consultation; sex; height; weight; type and age of onset of pubertal signs prompting consultation; Tanner stage for breast and pubic hair development; age at Tanner stage 2 breast development; testicular volume; presence or absence of menarche; bone age assessed by hand radiography; and serum hormone concentrations, including luteinizing hormone (LH), follicle-stimulating hormone (FSH), estradiol (E2), and testosterone.

The percentage of overweight was calculated as:

$$\frac{\left(\mathrm{measured}\;\mathrm{weight}\;-\;\mathrm{standard}\;\mathrm{weight}\;\mathrm{for}\;\mathrm{height}\right)}{\left(\mathrm{standard}\;\mathrm{weight}\;\mathrm{for}\;\mathrm{height}\right)}\times 100\text{\%}\;$$

The percentage of overweight was determined using age-, sex-, and height-specific standard body weight tables provided by the Japanese Society for Pediatric Endocrinology. In Japan, this metric is more commonly used than body mass index (BMI) to assess obesity in children. A value of 0% corresponds to the standard weight for a given age and height, whereas values ≥20% are conventionally considered indicative of obesity (9).

### 2.5 Statistical analyses

Statistical analyses were performed using R software (version 4.3.3). Annual incidence rates were calculated per 100,000 population using the number of children aged 0–14 years in Kanagawa Prefecture for each corresponding year as the denominator. Temporal changes in incidence and incidence rate ratios (IRRs) were initially assessed using Poisson regression models. Because overdispersion was observed, quasi-Poisson regression was applied. The Kruskal–Wallis test was used to compare median clinical parameters across the three time periods. A *p*-value <0.05 was considered statistically significant.

## 3 Results

Between January 2018 and December 2023, a total of 118 patients were diagnosed with CPP across the four participating hospitals. Of these, 94 (80.0%) were girls. The overall median age at presentation was 9.2 years [interquartile range (IQR): 8.1–10.1], the median BMI was 18.9 kg/m^2^ (IQR: 17.2–20.0), and the median percentage of overweight was 8.1% (IQR: −1.5–15.9). Baseline clinical characteristics are summarized in Table 1.

**Table 1:** Annual number of patients diagnosed with central precocious puberty (CPP) and their clinical characteristics.

	Pre-pandemic		Pandemic		Post-pandemic		Total
	2018	2019	2020	2021	2022	2023	2018–2023
*n*	5	8	12	27	32	34	118
Girls (%)	4 (80)	8 (100)	10 (83)	19 (70)	24 (75)	29 (85)	94 (80)
Chronological age at presentation (yr)	7.8 [7.6–9.0]	8.8 [8.1–9.2]	9.1 [7.9–10.0]	9.2 [8.3–10.0]	9.5 [8.4–10.2]	9.2 [8.1–10.2]	9.2 [8.1–10.1]
Body mass index (kg/m^2^)	18.9 [17.9–19.9]	18.0 [16.4–20.0]	18.7 [18.0–19.5]	19.1 [17.4–20.0]	18.8 [17.4–20.2]	18.4 [16.4–19.6]	18.9 [17.2–20.0]
Percentage of overweight (%)	14.6 [7.8–16.7]	6.2 [−1.7–22.4]	9.9 [4.8–13.0]	8.2 [2.2–16.2]	5.4 [−4.0–22.3]	7.2 [−3.5–13.6]	8.1 [−1.5–15.9]

Values are presented as median [interquartile range].

**Figure 1:**
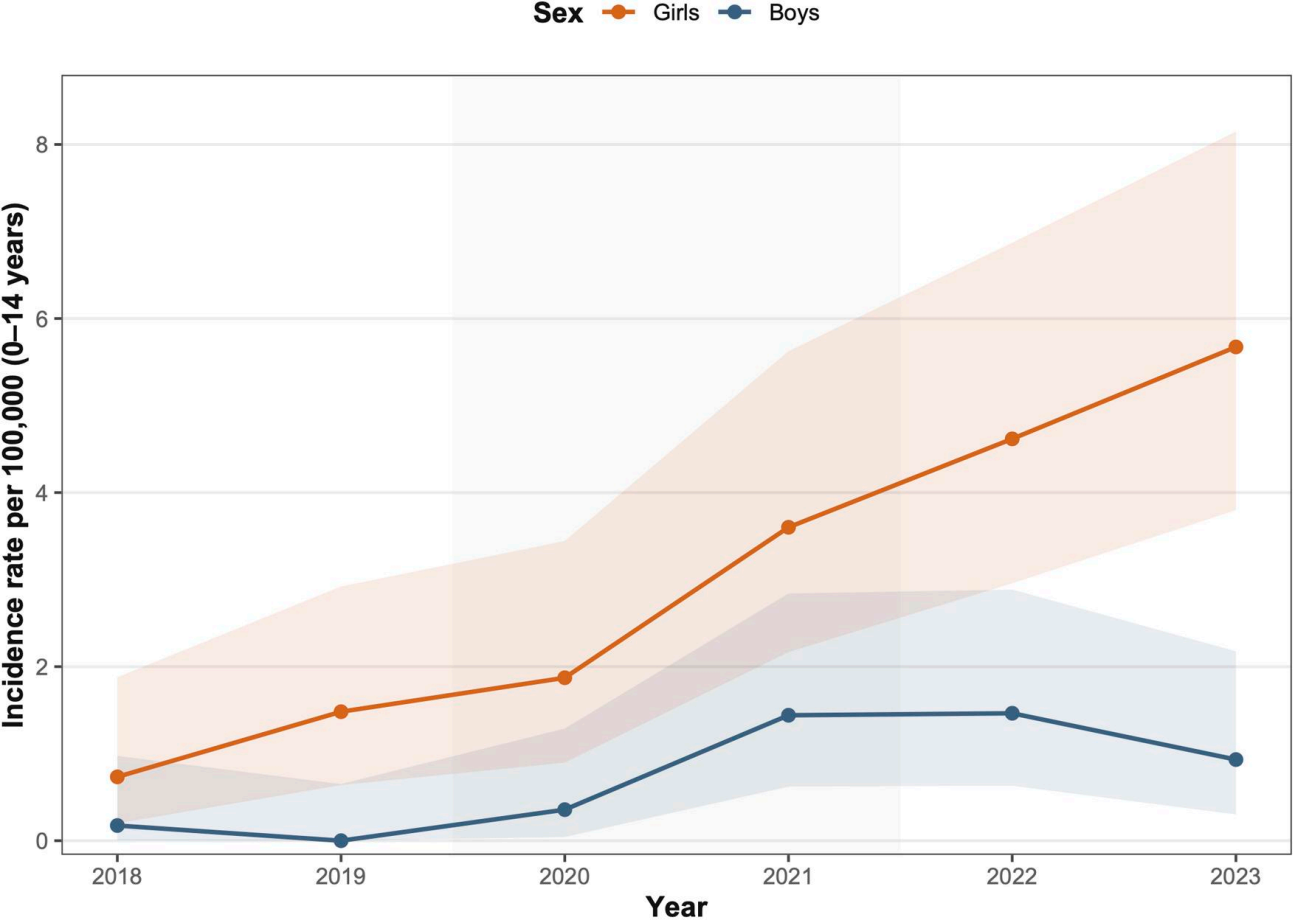
Annual incidence trends of central precocious puberty (CPP) in girls and boys, 2018–2023. Annual incidence of CPP per 100,000 children aged 0–14 years in Kanagawa Prefecture. Incidence rates were estimated using a quasi-Poisson regression model. Shaded ribbons represent 95% confidence intervals.

Annual CPP incidence rates per 100,000 population were calculated using the number of children aged 0–14 years in Kanagawa Prefecture as the denominator (Figure 1). In the quasi-Poisson regression model, the estimated annual increase in CPP incidence was 1.45-fold in girls [95% confidence interval (CI): 1.34–1.57] and 1.50-fold in boys (95% CI: 1.04–2.30), indicating a significant upward trend in both sexes over time.

**Figure 2:**
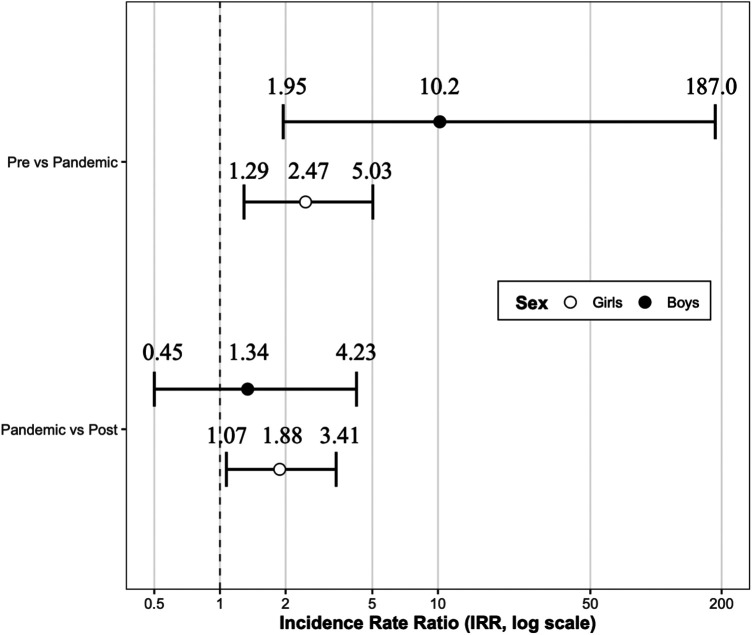
Incidence rate ratios across pre-pandemic, pandemic, and post-pandemic periods by sex. Forest plot showing incidence rate ratios (IRRs) comparing the pre-pandemic vs. pandemic periods and the pandemic vs. post-pandemic periods. Points indicate IRRs estimated using a quasi-Poisson regression model and horizontal bars represent the corresponding 95% confidence intervals.

To examine the effects of the COVID-19 pandemic, incidence rates were compared across pre-pandemic, pandemic, and post-pandemic periods (Figure 2). Among girls, the incidence of CPP increased significantly from the pre-pandemic to the pandemic period (IRR 2.47; 95% CI: 1.29–5.03; *p* = 0.0084). Although the IRR for boys was also statistically significant, the estimate was accompanied by wide confidence intervals, reflecting the limited number of cases and warranting cautious interpretation. When comparing the pandemic and post-pandemic periods, the IRR for girls remained elevated (IRR 1.88), but the difference was not statistically significant (*p* = 0.12). No significant changes were observed in boys across the same comparison (IRR 1.34; 95% CI: 0.45–4.23).

Clinical characteristics of female patients across the three defined periods are presented in Table 2. No significant differences were observed in age at presentation, age at onset of Tanner stage 2 breast development, interval from Tanner stage 2 to diagnosis, bone age, bone age advancement, chief complaint, or breast Tanner stage. Baseline hormone levels, including LH, FSH, and estradiol, were also comparable across groups.

**Table 2:** Clinical characteristics of girls with central precocious puberty across pandemic-related periods.

Variable	Pre-pandemic	Pandemic	Post-pandemic	*p*-value
*n*	12	29	53	
Chronological age at presentation (yr)	8.1 [7.3–8.7]	9.0 [7.8–9.3]	8.5 [7.7–9.7]	0.42
Chronological age at B2 (yr)	6.3 [6.0–6.9]	7.0 [6.5–8.0]	7.0 [6.5–7.5]	0.15
Time from B2 to diagnosis (yr)	1.1 [1.0–2.0]	1.0 [0.5–2.2]	1.4 [0.8–2.2]	0.28
Bone age (yr)	10.1 [8.5–10.4]	11.3 [10.1–12.0]	11.5 [10.6–12.0]	0.21
Bone age advancement (yr)	2.0 [1.3–2.5]	2.4 [1.9–2.7]	2.8 [2.1–3.2]	0.15
Basal luteinizing hormone (LH, mIU/mL)	1.6 [0.5–2.8]	2.2 [0.8–4.0]	2.5 [0.6–4.3]	0.53
Basal follicle-stimulating hormone **(**FSH, mIU/mL)	3.9 [2.6–5.3]	4.2 [3.6–5.3]	4.8 [3.4–5.6]	0.25
Basal estradiol **(**E2, pg/mL)	23.0 [13.7–40.0]	39.0 [25.9–50.0]	32.0 [24.0–47.2]	0.50
Chief complaint^a^ (%)				0.80
Breast enlargement	4 (33)	9 (31)	16 (30)	
Growth spurt	0 (0)	1 (3)	1 (2)	
Pubic or axillary hair development	3 (25)	2 (7)	8 (15)	
Genital bleeding	5 (42)	16 (55)	27 (51)	
Breast Tanner stage^a^ (%)				0.51
Stage 2	2 (17)	5 (17)	9 (17)	
Stage 3	4 (33)	14 (48)	24 (45)	
Stage 4	5 (42)	6 (21)	19 (36)	
Stage 5	1 (8)	1 (3)	0 (0)	
Percentage of overweight (%)	10.5 [−2.65–18.8]	8.8 [3.2–15.9]	10.4 [−1.7–16.0]	0.90

Pre-pandemic: 2018–2019; Pandemic: 2020–2021; Post-pandemic: 2022–2023.

Values are presented as median [interquartile range].

*p*-values were calculated using the Kruskal–Wallis test.

^a^ Due to missing data, denominators may differ from the total number of participants (n) for some variables.

Among boys (Table 3), no significant differences were detected in age at presentation, age at symptom onset, bone age, bone age advancement, chief complaint, testicular volume, or baseline FSH or testosterone levels. However, unlike the pattern observed in girls, LH levels at diagnosis differed significantly among the three periods (*p* = 0.021), with higher values during the pandemic compared with the post-pandemic period.

**Table 3:** Clinical characteristics of boys with central precocious puberty across pandemic-related periods.

Variable	Pre-pandemic	Pandemic	Post-pandemic	*p*-value
*n*	1	10	13	
Chronological age at presentation (yr)	10.9 [N/A]	10.5 [10.3–10.6]	10.5 [10.2–10.7]	0.58
Bone age (yr)	13.0 [N/A]	12.4 [12.2–12.9]	13.0 [12.5–13.5]	0.23
Bone age advancement (yr)	2.1 [N/A]	1.9 [1.8–2.5]	2.5 [2.0–2.8]	0.34
Basal luteinizing hormone (LH, mIU/mL)	1.9 [N/A]	2.1 [1.6–3.5]	1.1 [1.0–2.1]	0.021^b^
Basal follicle-stimulating hormone (FSH, mIU/mL)	1.7 [N/A]	3.3 [3.0–3.7]	3.5 [3.2–4.1]	0.35
Basal testosterone (T, ng/mL)	2.4 [N/A]	2.1 [1.2–3.1]	2.7 [1.3–4.9]	0.78
Testicular volume (mL)	15 [N/A]	10 [8–15]	12 [9–15]	0.78
Percentage of overweight (%)	17.6 [N/A]	7.1 [−0.7–11.5]	1.9 [−7.7–5.4]	0.11
Chief complaint^a^ (%)				0.23
Testicular enlargement	0 (0)	0 (0)	0 (0)	
Growth spurt	0 (0)	0 (0)	0 (0)	
Pubic or axillary hair development	1 (100)	2 (20)	5 (38)	
Voice change or facial hair growth	0 (0)	8 (80)	7 (58)	

Pre-pandemic: 2018–2019; Pandemic: 2020–2021; Post-pandemic: 2022–2023.

Values are presented as median [interquartile range]. Because only one patient was included in the pre-pandemic group, interquartile ranges are shown as not available (N/A).

*p*-values were calculated using the Kruskal–Wallis test.

^a^ Owing to missing data, denominators may differ from the total number of participants (*n*) for some variables.

^b^ Statistically significant (*p* < 0.05).

With regard to adiposity, no statistically significant differences in percentage of overweight were identified across the three periods for either sex. Nevertheless, median percentages of overweight were consistently above 0% in all groups, suggesting a persistent trend toward higher adiposity among children diagnosed with CPP.

## 4 Discussion

In this multicenter retrospective study, we observed a clear increase in newly diagnosed CPP among girls during the COVID-19 pandemic compared with the pre-pandemic period, reaching statistical significance in the quasi-Poisson model. When comparing the pandemic and post-pandemic periods, the IRR in girls remained elevated, suggesting a persistence of increased incidence beyond the acute pandemic phase.

In contrast, interpretation of temporal changes in CPP incidence among boys is substantially limited by the small number of cases. Although the quasi-Poisson analysis yielded a numerically large IRR when comparing the pre-pandemic and pandemic periods, the extremely wide confidence intervals indicate considerable statistical uncertainty. Accordingly, these findings should be interpreted with caution and do not permit firm conclusions regarding true population-level changes in CPP incidence among boys.

Previous studies have generally reported stable CPP incidence in boys during the pandemic (7, 10, 11). While our data suggest a possible increase, this observation should be regarded as hypothesis-generating rather than confirmatory. Emerging evidence indicates that environmental and metabolic factors, including adiposity, may influence pubertal timing in boys more than previously recognized (12); however, larger population-based studies are required to determine whether pandemic-related lifestyle changes had a meaningful impact on male CPP incidence.

It should be emphasized that the terms “pandemic” and “post-pandemic” in this study represent pragmatic timeframes defined by public health measures in Japan, rather than discrete biological or societal endpoints. To our knowledge, this is the first multicenter study in Japan to document sex-specific temporal patterns in CPP incidence, and our findings are broadly consistent with international multicenter studies (13–15) and recent meta-analyses (16).

Lifestyle changes during the pandemic—including increased screen time, reduced physical activity, shortened sleep duration, and heightened psychological stress—have been widely discussed as potential contributors to earlier pubertal onset (17, 18). In the present study, no statistically significant differences in the percentage of overweight were observed across the three time periods for either sex. Nevertheless, median values consistently exceeded 0% in all groups, indicating a general tendency toward higher adiposity among children diagnosed with CPP.

Importantly, these findings should not be interpreted as evidence of a causal relationship between overweight and the observed increase in CPP incidence. Rather, the consistently elevated percentage of overweight likely reflects a broader background context in which pandemic-related lifestyle changes occurred. National surveillance data from Japan have similarly reported increases in pediatric overweight during the pandemic (19), supporting the notion that population-level shifts in adiposity occurred during this period. Collectively, these observations suggest that overweight may represent one of several coexisting environmental or metabolic factors associated with CPP, rather than a direct driver of incidence changes.

The most pronounced increase in CPP incidence was observed during the pandemic, particularly among girls, where the rise from pre-pandemic levels was statistically significant and appeared to persist into the post-pandemic period. No such persistence was observed among boys, suggesting potential sex differences in long-term susceptibility to lifestyle modifications introduced during the pandemic.

This pattern contrasts with reports from other regions describing normalization or decline in CPP incidence following the easing of pandemic-related restrictions (20, 21). Differences in the duration and intensity of lifestyle changes—such as prolonged alterations in physical activity, screen exposure, and daily routines—may partly explain these discrepancies. In several European cohorts, a reduction in CPP diagnoses was observed following the lifting of strict lockdown measures, suggesting a partial normalization of environmental exposures. Notably, the public health response in Japan differed from that in many other countries, as strict lockdown measures were not implemented and social restrictions were relatively moderate. As a result, behavioral adaptations introduced during the pandemic may have been less abrupt but more sustained, potentially contributing to the continued elevation in incidence observed in our cohort.

Furthermore, previous studies have demonstrated a gradual decrease in the age of pubertal onset in girls over recent decades (22). When considered alongside this secular trend, our findings raise the question of whether Japan's current diagnostic criteria for CPP—which have remained unchanged since 2007—adequately reflect contemporary patterns of pubertal development.

This study has several limitations. First, although data were collected from four tertiary hospitals, the number of male patients was relatively small, limiting the statistical power of subgroup analyses. Second, multiple comparisons were performed without formal adjustment; therefore, findings from secondary analyses—particularly hormone measurements in boys—should be interpreted cautiously. Third, bone age assessment, Tanner staging, and determination of symptom onset were performed by different clinicians across institutions, potentially introducing inter-observer variability. Fourth, despite being the largest multicenter investigation of CPP in Japan to date, the retrospective design and reliance on electronic medical records may have resulted in incomplete capture of certain clinical variables. Finally, population-based incidence estimates assume stable hospital catchment areas; however, healthcare-seeking behavior and referral patterns may have changed during the COVID-19 pandemic. Delayed consultations, reduced access to primary care, or increased referrals to tertiary centers after easing of restrictions may have contributed to the observed increase in newly diagnosed cases. Accordingly, the reported trends should be interpreted with caution, as changes in healthcare utilization may have partially influenced case numbers in addition to any underlying changes in disease incidence.

## 5 Conclusion

This multicenter study demonstrated a significant increase in the incidence of CPP among girls during the COVID-19 pandemic, with possible continuation into the post-pandemic period, whereas trends among boys were less certain. Although not all comparisons reached statistical significance, the overall pattern is consistent with international observations and suggests that the pandemic may have accelerated pre-existing shifts toward earlier pubertal onset. These findings underscore the importance of continued nationwide surveillance and careful evaluation of whether current diagnostic criteria for CPP remain appropriate in contemporary clinical practice.

## Data Availability

The raw data supporting the conclusions of this article will be made available by the authors, without undue reservation.
